# Diet and ileostomy: a qualitative comparison of patient and healthcare professional perspectives in the United Kingdom and Australia

**DOI:** 10.1007/s00394-026-04036-1

**Published:** 2026-06-15

**Authors:** Niamh Magee, Ellen E. A. Simpson, Helen McCarthy, Pauline Douglas, Erika Rosbotham, L. Kirsty Pourshahidi, James Davis, Martin Veysey, Nenad Naumovski, Chris I. R. Gill

**Affiliations:** 1 Nutrition Innovation Centre for Food and Health (NICHE), Ulster University, Coleraine, UK; 2 School of Psychology, Ulster University, Coleraine, Northern Ireland UK; 3 Institute for Health and Sport, Victoria University, P.O. Box 14428, Melbourne, VIC 8001 Australia; 4 School of Engineering, Ulster University, Belfast, BT15 1ED Northern Ireland, UK; 5 School of Medicine and Psychology, Australian National University, Canberra, ACT 2605 Australia; 6 Food, Chemical & Biotechnology Cluster, Singapore Institute of Technology, Singapore, 828608 Singapore

**Keywords:** Ileostomy, Healthcare professional, Dietary advice, Stoma, Qualitative

## Abstract

**Purpose:**

Ileostomy formation requires major dietary adjustments to prevent complications and maintain nutritional status, yet dietary advice is often inconsistent, and evidence guiding best practice remains limited. This study explored how people living with an ileostomy (ileostomates) and healthcare professionals (HCPs) experience and support dietary management, uniquely comparing UK and Australian perspectives.

**Method:**

Fifty semi-structured interviews were conducted with ileostomates (*n* = 26) and HCPs (*n* = 12 stoma care nurses, *n* = 12 dietitians) across the UK and Australia. Interviews were recorded, transcribed and analysed using thematic content analysis guided by the Social Ecological Model (SEM).

**Results:**

Seven main categories emerged, spanning intrapersonal, interpersonal, community, institutional and public policy factors. Ileostomates reported persistent dietary restriction, fear of food bolus blockages, and reliance on online forums when HCP follow up was limited. HCP transcripts highlighted variations in practice, constrained caseloads, and gaps in evidence underpinning dietary guidance. Country-specific differences were observed, with Australian ileostomates emphasising hydration and geographical barriers. HCPs in the UK highlighted reinforcement of advice from a stoma nurse or dietitian as being central to dietary reintroduction, whereas their Australian counterparts prioritised a patient-led approach.

**Conclusion:**

Findings reveal systemic gaps in dietary support across both countries and highlight the need for individualised support, improved professional training, and integration of digital or telehealth solutions to enhance accessibility and continuity of care.

**Supplementary Information:**

The online version contains supplementary material available at 10.1007/s00394-026-04036-1.

## Introduction

 An estimated 200,000 people in the UK, and a further 50,000 in Australia, live with a stoma [1, 2]. Of these, approximately 36% have an ileostomy, equating to 90,000 individuals across both countries [3]. Ileostomy surgery involves externalisation of the ileum onto the abdominal wall with or without removal or resection of the ileocecal valve and the large intestine, allowing for diversion of digested material into a disposable pouch. This procedure is typically required due to refractory inflammatory bowel disease (IBD) or as part of cancer treatment [4]. Living with an ileostomy requires significant physiological and psychosocial adaptations [5], including dietary changes in response to an altered digestive system [6], maintenance of electrolyte balance, and to prevent postoperative complications i.e. stomal blockages [7].

Despite the vital role of diet in surgical recovery and stoma management, provision of formal dietary advice, often by a stoma care nurse (SCN) or dietitian, can be inconsistent with reported gaps in guidance [8]. Although fibre reintroduction is generally encouraged to support a normal, varied diet [6], being “advised to avoid” certain items in the early postop period will inevitably influence long-term eating behaviours [9]; the continuation of a low fibre diet is reported widely in the literature [10]. Ongoing dietary restriction has been associated with reduced overall and diet-related quality of life (QoL) [11], yet evidence guiding dietary management for ileostomates remains limited. These challenges highlight an urgent need to better understand how dietary advice is delivered and interpreted in both clinical and in informal contexts.

Differences in health systems may further shape dietary support, and cross-national comparisons can identify opportunities for improvements in policy and subsequent patient outcomes [12]. The tax-funded UK National Health Service provides care which is free and based on clinical need [13], whereas Australia operates a hybrid public-private system, underpinned by universal health insurance (Medicare) which subsidises the cost of medical services [14]. Australia’s vast geography impacts the availability of tertiary care in rural and remote regions [15], creating difficulty for those living in these regions who need specialist services. Spatial inequalities in the UK health services are still evident, albeit less extreme in nature [16], and both countries face shared pressures, including aging populations and increasing pressure to reduce healthcare costs [17].

Consequently, comparing perspectives across patients and HCPs, within and across healthcare systems, can provide an understanding of dietary support for people living with an ileostomy. To that end, the aims of this study were to establish and compare experiences of (1) adult ileostomates provided with dietary advice/support, and (2) the healthcare professionals (HCPs) (SCNs, dietitians) providing dietary advice/support across UK and Australian contexts.

## Methods

This study follows the Standards for Reporting Qualitative Research guidelines [18].

### Theoretical framework

**Fig. 1:**
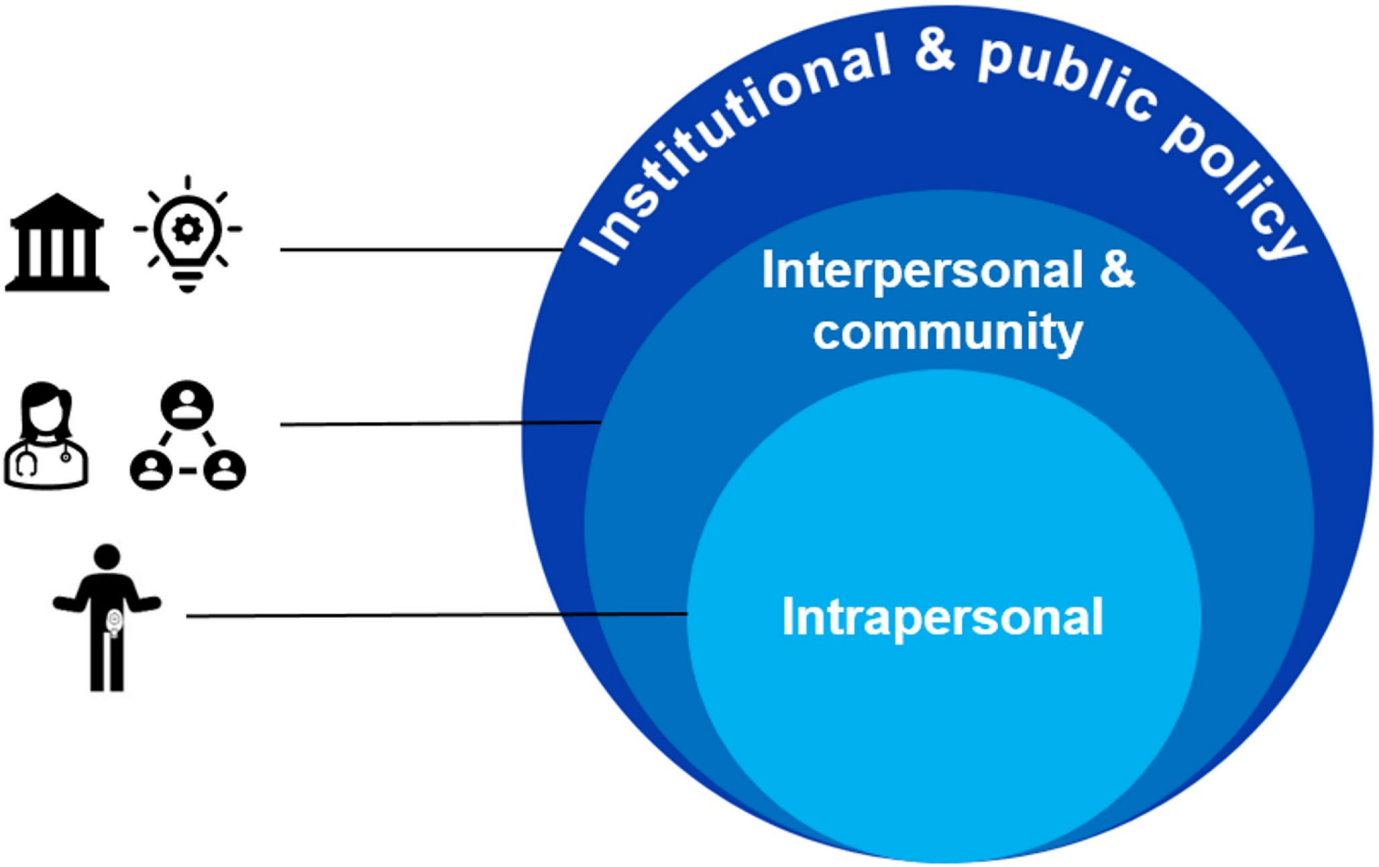
Theoretical framework adapted from Bronfenbrenner’s (1979) Social Ecological Model

Given the significant dietary adaptations required following ileostomy surgery, a comprehensive framework is essential to understand the multiple influences on care experiences. A qualitative descriptive approach was adopted, underpinned by a pragmatic research paradigm and guided by the Social Ecological Model (SEM). The SEM recognises that experiences are shaped by a range of intersecting factors [19]. For this study, these were consolidated into three levels: (1) intrapersonal relating to ileostomates’ needs and experiences, (2) interpersonal and community relating to support mechanisms, and (3) institutional and public policy relating to overarching systems and policies which dictate clinical practice (Figure 1).

### Study design

Semi-structured interviews were conducted to ensure consistency across participants whilst allowing flexibility to explore individual perspectives [20]. Topic guides (**Table ****S1**) were underpinned by the SEM and co-created with input from patients (*n* = 3) and HCPs (*n* = 1 dietitian, *n* = 1 SCN); feedback was collated, and the guides were further refined by NM, HMcC and PD. Ethical approval was granted by Ulster University (FCBMS-24-011-C) with reciprocal approval from Victoria University, in accordance with the Declaration of Helsinki (2024).

### Participants and recruitment

Two participant groups were included: (1) ileostomates (> 18 years, > 8-weeks postop) residing in the UK or Australia, (2) HCPs (SCNs and dietitians) regularly working with ileostomates and practising in the UK or Australia. Purposive sampling ensured variation in key characteristics including age, gender, time since surgery (ileostomate), professional role and experience (HCP). Recruitment was via social media (Facebook, Instagram), peer support groups (e.g. Ileostomy Association), and professional bodies (e.g. Association of Stoma Care Nurses). Eligible participants were identified via screening questionnaire and provided with an information sheet outlining the purpose of the study, before providing their written informed consent.

### Data collection

Prior to interview, participants completed a survey *via* REDCap (v14.5.32), indicating ileostomy history or professional experience where applicable. One-on-one interviews took place online *via* Microsoft Teams (v25306.804) or in-person interviews were offered where feasible. UK interviews were conducted between May – November 2024 and Australian interviews between March – June 2025. Topic guides were administered flexibly to facilitate discussion. All interviews were conducted by NM to ensure consistency, audio and/or video recorded and transcribed verbatim. No relationship was established with participants prior to taking part.

### Data analyses

Descriptive statistics were used to present demographic data (IBM SPSS Statistics v.29); categorical data presented as n (%) and mean ± SD for numerical data. Interview transcripts were reviewed for accuracy, anonymised and imported into data analysis software (NVivo 14.23.3). Thematic content analysis followed Braun and Clarke’s six-step approach, using inductive and deductive coding [21], with the deductive component informed by the SEM, alongside inductive coding to capture emergent insights. Two researchers (NM, EEAS) independently coded a subset of the transcripts (*n* = 7 ileostomate, *n* = 6 HCP) achieving 85.7% agreement [22]. Discrepancies were resolved through discussion to form the codebook [23], which was systematically applied to the remaining data. Sample size was guided by the principle of data saturation, with interviews continuing until no new codes or ideas emerged within each group, indicating an adequate sample was reached – consistent with established qualitative research practice [24]. Codes were grouped into categories and subcategories according to the SEM (Fig. 1), which were further analysed for commonalities and refined as appropriate. Through discussion, consensus was reached on the assignment and definition of each category and its subcategories (100% agreement). Quotations were extracted from the transcripts for illustrative purposes.

### Researcher characteristics and reflexivity

The research team are experienced in nutrition and psychology research for people living with a stoma. PD and HMcC also have clinical experience as gastrointestinal dietitians. To build positionality, NM maintained reflexive notes during data collection and analysis; these reflections were continually discussed with EEAS.

## Results

### Participant characteristics

All interviews were conducted *via* Microsoft Teams, except one face-to-face interview (Ulster University, Coleraine, Northern Ireland). Data saturation was reached after *n* = 13 ileostomate and *n* = 12 HCP interviews in both UK and Australian cohorts. Characteristics of UK and Australian participants were comparable for both groups (Table 1).

**Table 1:** Participant characteristics

Characteristic	Ileostomate (*n* = 26)		HCP (*n* = 24)	
	*UK (n* = 13)	*AUS* (*n* = 13)	*UK* (*n* = 12)	*AUS* (*n* = 12)
Gender (*n*, %)				
Male	6 (46.2)	6 (46.2)	1 (8.3)	1 (8.3)
Female	7 (53.8)	7 (53.8)	11 (91.7)	11 (91.7)
Age (years, mean ± SD)	49.1 ± 14.7	56.2 ± 15.0	38.5 ± 9.9	42.0 ± 11.1
Reason for ileostomy (n, %)				
Ulcerative Colitis	5 (38.5)	3 (23.1)	N/A	
Cancer treatment	2 (15.4)	4 (30.8)		
Crohn’s Disease	3 (23.1)	5 (38.5)		
Diverticulitis	1 (7.7)	1 (7.7)		
Other	2 (15.4)^a^	0 (0.0)		
Time since surgery (years, mean ± SD)	8.3 ± 8.0	10.7 ± 14.9	N/A	
Other dietary restrictions (n, %)				
Yes	0 (0)	3 (23.1)^b^	N/A	
No	13 (100)	10 (76.9)		
Job role (n, %)	N/A			
Dietitian			7 (58.3)	5 (41.7)
Stoma care nurse			5 (41.7)	7 (58.3)
Job setting (n, %)	N/A			
Acute			9 (75.0)	8 (66.7)
Community			3 (25.0)	4 (33.3)
Time spent in current role (n, %)	N/A			
6–12 months			0 (0.0)	1 (8.3)
1–3 years			5 (41.7)	3 (25.0)
4–5 years			2 (16.7)	1 (8.3)
5–10 years			2 (16.7)	3 (25.0)
> 10 years			3 (25.0)	4 (33.3)
Interview duration (min, mean ± SD)	21.7 ± 6.6	26.8 ± 7.6	23.2 ± 4.3	28.6 ± 7.4

HCP, health care professional; AUS, Australia; SD, standard deviation

^a^*n*=1 ileostomy due to slow colon transit with toxic megacolon; *n* = 1 ileostomy due to complication following separate surgery

^b^*n*=1 gluten-free, dairy-free and vegetarian diet; *n* = 2 vegetarian diet

### Thematic content analyses

**Fig. 2:**
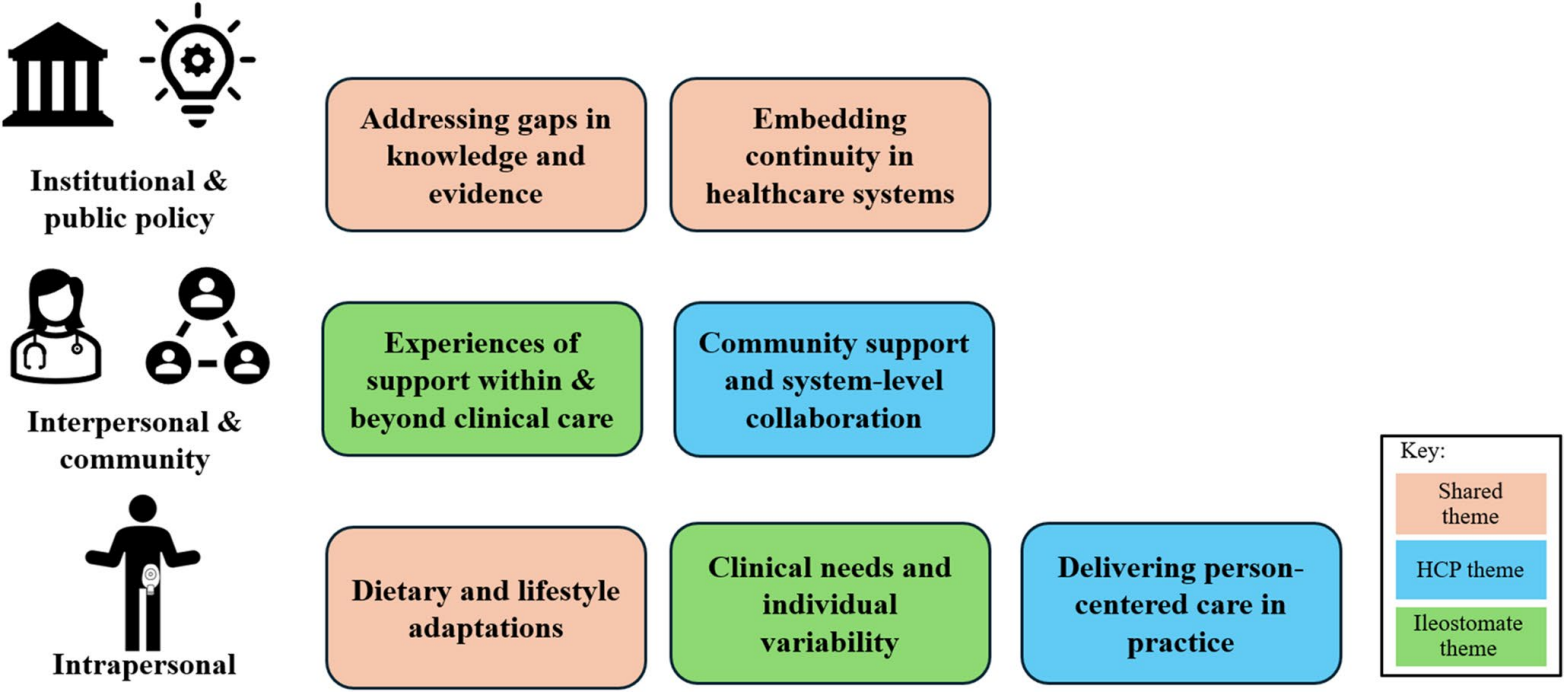
Main categories identified from ileostomate, and HCP transcripts mapped onto the Social Ecological Model

Analysis of UK and Australian transcripts revealed 7 main categories (Fig. 2); 3 common to both groups, 2 unique to patient experiences, and 2 unique to HCP experiences. Each category captures a broader domain of experience, with subcategories representing specific aspects within these domains, mapped onto one of the three levels of the SEM. Country specific differences are observed in the subcategories contained within each category; unique subcategories which emerged from each group (ileostomate and HCP, UK and Australia) are outlined alongside illustrative quotes in Table 2 (intrapersonal), Table 3 (interpersonal and community) and Table 4 (institutional and public policy). Prevalence of subcategories across participant groups is summarised in Table **S2**.

### Intrapersonal

#### Category 1: dietary and lifestyle adaptations (shared)

Dietary and lifestyle changes were a central feature of participant experience; subcategories reflect how various overlapping factors, including *dietary restriction*, *symptom management* and *patient understanding*, can interact to shape dietary management. Ileostomates reported dietary restriction after surgery, particularly adherence to a low fibre diet. This was noted as routine postop advice by HCPs, although the suggested timeframe for following low fibre advice differed between professionals:

*“…as a standard*,* they’re all just provided with low fibre advice*,* first 6 to 8 weeks.”* [H06, dietitian, UK].

*“…when we g*et *around to that 12-week mark…we might start reintroducing some of the foods.”* [H17, SCN, Australia].

Australian HCPs placed greater emphasis on avoiding long-term restriction, and both groups noted that many patients continued limiting fibre beyond the recommended period, with negative consequences.

Ileostomates (UK and Australia) described how insufficient contact time with HCPs during inpatient admission and limited or lack of follow-up prompted them to adopt a *self-directed trial and error* approach. While this increased confidence and dietary independence for some, others developed more persistent or severe restrictions. For UK participants, *symptom management* – particularly fear of blockages – further exacerbated *dietary restriction* and hindered enjoyment of food. Both groups recognised long-term impacts of *living with dietary change* on eating in social settings and timing of meals. The psychosocial challenges of undergoing this surgery were recognised by HCPs, who noted their importance in supporting patients through a major life change. Indeed, ensuring *patient understanding* of dietary advice was considered central to reducing long-term restriction, highlighting the importance of ongoing support from HCPs. Australian HCPs additionally emphasised education on dehydration risk, mirroring Australian ileostomates who highlighted their concerns surrounding output and hydration management.

#### Category 2: Clinical needs and individual variability (ileostomate)

Variation in dietary management was influenced by clinical context, as outlined by the subcategories emerging from UK and Australian transcripts. Ileostomates recognised that factors determining *need for surgery* (e.g. the type/severity of the underlying condition(s); elective or emergency surgery) directly influenced later dietary management. *Surgical recovery and postoperative complications* also played a significant role, with many reporting bland diets, delayed appetite return, and the impact of postoperative ileus on ileostomates’ confidence when reintroducing foods. *Undernutrition and weight management* were major concerns across both countries, with some requiring parenteral nutrition or oral supplements to maintain nutritional status. While dietetic support was valued, participants noted that a focus on nutrition support meant that ileostomy-specific advice was overlooked in some instances.

Australian ileostomates uniquely described difficulties managing *special dietary requirements*, particularly those who were following a vegetarian diet and relied on high fibre foods as a source of protein. Moreover, Australian participants uniquely linked dietary intake to *pouch leakage and peristomal skin complications*, describing how these further complicated dietary choices.

#### Category 3: Delivering person centred care in practice (HCP)

Within this category, each subcategory reflects different operational aspects of person-centred practice. HCPs from both countries described the importance of integrating needs in their practice, this involved consideration of clinical needs, postop stage and personal preferences and for Australian HCPs, this also included consideration of social and cultural factors. When delivering person centred care, UK HCPs highlighted the need for *reassurance and reinforcement of advice* to ease patients’ anxiety around eating, whereas Australian HCPs emphasised *patient-led stepwise support* tailored to individual readiness and preferences.

**Table 2:** Summary of categories, subcategories and illustrative quotes relating to interpersonal factors

Ileostomate		HCP	
Dietary & lifestyle adaptations		Dietary & lifestyle adaptations	
*Dietary restriction* UK, AUS	*“I’ve pretty much decided that* [fibrous foods], *they won’t be a part of my diet*,* despite the fact that they used to be.”* [S17, AUS] *“I take what I know I can take and stay clear of anything else.”* [S01, UK]	*Dietary restriction* UK, AUS	*“You need to eat a balance of everything*,* and food restriction is not good…* [patients] *are trying to control their stoma with what they’re eating and drinking. And it’s not the right thing to do.”* [H13, SCN, AUS]
*Self-directed trial & error* UK, AUS	*“So*,* a lot of it was self-led*,* and I just tried to balance things out… a lot of it was just done by myself.”* [S10, UK] *“It’s just trial and error*,* which can be very*,* very tiring.”* [S24, AUS]	*Providing support during a major life change* UK, AUS	*“I think that’s also really good to engage from a counselling point of view because you could pick up whether or not some of these issues are because*,* you know*,* there’s actually issues with their stoma or… they’re just not coping with the fact that they actually have a stoma.”* [H18, dietitian, AUS]
*Living with dietary change* UK, AUS	*“I try not to talk while I’m eating*,* otherwise it just goes into the bag*,* so I’m not good company at the dinner table normally.”* [S16, AUS] *“If I keep on chewing…then you don’t have to worry. But… it takes the flavour away and the enjoyment.”* [S13, UK]	*Fear & uncertainty* UK, AUS	*“Some patients g*et *to the point where they’re too scared to try. That’s not good because the longer you leave it*,* the harder it gets.”* [H10, SCN, UK]
*Symptom management* UK	*“Broccoli*,* I love. But I have to be careful of it as well*,* because one flor*et *of broccoli nearly - that’s the only time I had a really bad blockage*,* and it was one wee* [little] *flor*et *of broccoli”* [S02, UK]	*Patient understanding* UK, AUS	*“They haven’t had a bowel transplant. And I keep saying this to patients*,* it’s still your body… if it didn’t like that before*,* it may not like it now*,* but if you’re used to eating certain foods…”* [H02, SCN, UK]
*Output & hydration* AUS	*“For me it’s more making sure the fluid intake is*,* is enough and then if it’s I’m still putting out a lot. Make sure that I’m getting in the electrolytes and the salt.”* [S18, AUS] *“I personally just have to make certain adjustments like I have to keep in mind I have to empty my bag a little bit more often.”* [S015, AUS] *“It’s just a liquid output after I eat them. But if I eat any vegetable or fruit*,* well*,* I can’t eat fruit…”* [S22, AUS]		
Clinical needs & individual variability		Delivering person centred care in practice	
*Need for surgery* UK, AUS	*“Luckily for me*,* I already knew about a stoma bag*,* and I already had a pretty crap life before*,* in a sense*,* where*,* like*,* you know*,* you’re always having to worry about your diet…”* [S15, AUS]	*Integrating needs* UK, AUS	*“You’re dealing with social issues on top of having an ileostomy… you’re having ileostomy patients who are alcoholics*,* diabetics*,* very poor health choices*,* and our dietitian was expected to cover everyone.”* [H23, SCN, AUS]
*Surgical recovery & postop complications* UK, AUS	*“…that was quite*,* sort of like*,* intense and quite traumatic for me. So*,* I think that fear of ‘oh if I eat something and I have a blockage and if I have to have an NG drain again*,*’ like I don’t want that*,* you know?”* [S08, UK] *“And I was… very*,* very paranoid about blockages and things like that because of what I’ve experienced… the pain level that I experienced was horrific*,* so I was very worried about the possibility of blockage.”* [S25, AUS]	*Reassurance & reinforcement of advice* UK	*“I g*et *that feedback from them at times*,* you know*,* that that they feel more reassured*,* having been in to see somebody. I think they need that… if they don’t g*et *the reassurance then it’s it’s much more difficult.”* [H03, dietitian, UK]
*Undernutrition & weight management* UK, AUS	*“The dietitian was mostly just there for the weight loss and stuff actually.”* [S15, AUS]	*Patient-led stepwise support* AUS	*“I tend to always be really guided by the patient. So sometimes they just want*,* you know*,* time to themselves… Sometimes people really sort of like to go through the resource with me and go through each point and expand on everything.”* [H15, dietitian, AUS]
*Special dietary requirements* AUS	*“My problem being a veggie. You know*,* if I wasn’t a veggie*,* it’d been a lot better”* [S16, AUS].		
*Pouch leakage & PSCs* AUS	*“And especially with chemo*, [the PSC] *got worse. There was the burning of skin and*,* and so food and everything is sometimes a nightmare really*,* to be honest.”* [S22, AUS] *“The biggest problem I have is I get a lot of ulcerations by certain foods around the ileostomy.”* [S24, AUS]		

HCP, health care professional; SCN, stoma care nurse; AUS, Australia; PSC, peristomal skin complication

### Interpersonal and community

Subcategories within this level illustrate how dietary management is shaped by the availability, accessibility and perceived credibility of multiple support systems on an intrapersonal and community level.

#### Category 4: Experiences of support within and beyond clinical care (ileostomate)

The *value of HCP* support was emphasised by ileostomates from the UK and Australia; support from professionals shaped dietary experiences within clinical care and promoted confidence in dietary reintroduction beyond the postop period. However, access to care for Australian ileostomates was notably affected by *geographical barriers*, with those in regional and remote areas struggling to access specialist services.

Beyond formal clinical care, *family support* was highly important for dietary adaptation during the initial postop period and many UK and Australian ileostomates relied on *peer experience and online forums*, to supplement HCP support in the longer term, albeit views on the reliability of such advice were mixed.

#### Category 5: Community support and system-level collaboration (HCP)

Across UK and Australian transcripts, HCPs highlighted *interprofessional working* as an important element of delivering patient care, both between dietetic and stoma care teams, as well as other healthcare staff. Related to interprofessional working, HCPs emphasised the importance of consistent messaging when delivering dietary advice across the patient pathway. Inconsistent or conflicting advice was viewed as detrimental to patients’ confidence in professionals, subsequently influencing implementation of dietary advice. Further, hospital meals were frequently reported as lacking in flavour and variety, highlighting the *provision of hospital food* as a considerable barrier to postop dietary intake.

Aligning with ileostomate experiences, the subcategory *peer experience and online forums* emerged from interviews with UK professionals. HCPs noted that these sources presented an opportunity for mixed messages and confusion, cautioning use of online resources. Similarly, UK HCPs described *family support* as a potential facilitator and barrier to effective care, highlighting the importance of involving them in education and decision-making. Similarly, the subcategory g*eographical barriers* was identified by Australian HCPs working in regional areas, echoing the experiences of the Australian patient group. Here, telehealth services were noted as an efficient method of combating such challenges.

**Table 3:** Summary of categories, subcategories and illustrative quotes relating to interpersonal and community factors

Ileostomate		HCP	
Experiences of support within & beyond clinical care		Community support and system-level collaboration	
*Value of HCP support* UK, AUS	*“They did give me the confidence to*,* to try the brown bread and*,* and other things… I was so frightened beforehand.”* [S04, UK]	*Interprofessional working* UK, AUS	*“We have quite good links with our stoma nurses*,* so they wouldn’t hesitate to g*et *in touch with us.”* [H05, dietitian, UK]
*Geographical barriers* AUS	*“…as far as specialists and like*,* like I said*,* stoma nurses. Yeah*,* you do generally have to travel some distance. So*,* like my surgeon*,* he’s like 3 1/2 hours away from me.”* [S24, AUS]	*Geographical barriers* AUS	*“Anybody who’s sort of remote*,* or far away*,* or outside of the* [Australian Capital Territory], *we will definitely offer*,* will be a lot of telephone… I’m not going to make a patient drive 4 hours here and 4 hours back*,* just for a dietetic appointment.”* [H18, dietitian, AUS]
*Peer experience & online forums* UK, AUS	*“You’re getting advice sometimes on these Facebooks*,* from ordinary people*,* they’re not doctors*,* you know*,* and they’re not medical people… it’s hard to know whether you trust them or not.”* [S13, UK]	*Peer experience & online forums* UK	*“So*,* they’re taking it from*,* you know*,* from I don’t know from TikTok or other aunties*,* uncles’ mother*,* that has a stoma*,* you know*,* and things like that. And I always say to them that*,* you know*,* you*,* they*,* that may be applicable to them*,* but maybe not to you.”* [H08, SCN, UK]
*Family support* UK, AUS	*“I would have really struggled with*[out] *having a sympathetic wife*,* to cope… No*,* no. I don’t think I would have coped. I wouldn’t have coped.”* [S16, AUS]	*Family support* UK	*“…and sometimes it’s family that can be a help or a hindrance if they’re going*,* ‘but they can’t have that*,*’ you’re trying to explain to them as well why they can have it*,* and it’s trying to g*et *them all to work together.”* [H05, dietitian, UK]
		*Importance of consistent messaging* UK, AUS	*“They are told about every different message from different people*,* the doctors*,* stoma care nurse*,* the nurse itself*,* and me giving them another piece of advice… They kind of feel overwhelmed and sceptical about how useful that’s gonna be. So sometimes that shakes my confidence too.”* [H22, dietitian, AUS]
		*Provision of hospital food* UK, AUS	*“I think it’s just the food… It’s not very good. It’s not very palatable. They don’t find there’s much flavour*,* UM*,* I think then they g*et *fed up just eating the same thing all the time… they’re not getting a great variety.”* [H07, SCN, UK]

HCP, health care professional; SCN, stoma care nurse; AUS, Australia

### Institutional and public policy

#### Category 6: Embedding continuity in healthcare systems (shared)

Continuity of dietary support was shaped by system-level factors, incorporating subcategories relating to early education, follow-up provision and system constraints. Ileostomates in both countries described feelings of uncertainty around dietary expectations in the lead up to surgery, emphasising the need for early education and continued follow-up to support dietary adjustment. From the HCP perspective, regular contact in the early postop period was viewed as critical to patients’ trust in professionals and encouraging dietary reintroduction, however it was noted that this was not always feasible due to system pressures. *Managing caseload capacity* was impeded by several *system constraints*, namely frequent staff turnover, funding limitations and, for Australian HCPs specifically, a s*hortage of specialist services* in regional and remote areas was a barrier to delivering comprehensive dietary support.

UK ileostomates reported difficulty accessing ongoing support after the initial recovery period. Although they acknowledged that resources and expertise exist, they felt that *signposting* and accessibility within the healthcare system were inadequate. Similarly, Australian ileostomates described challenges in navigating online information and expressed scepticism about the reliability of resources. Participants reported needing to consult multiple sources to piece together a complete understanding of dietary management, highlighting the need for *trusted and comprehensive information.*

#### Category 7: Addressing gaps in knowledge and evidence (shared)

Gaps in knowledge and evidence underpinning dietary care were evident across both groups, encompassing subcategories relating to professional knowledge, variability in advice and limitations in the evidence base. Ileostomate and HCP participants in both countries identified gaps in professionals’ understanding of ileostomy management, which patients felt limited the quality of dietary guidance. *Improving HCP knowledge* was also a priority for HCPs, who noted the challenges of working in a specialist area, emphasising the need for continuous learning, regardless of clinical experience. Moreover, HCPs also highlighted the need to address knowledge gaps amongst colleagues who were less experienced in this area. Underpinning professional knowledge, patients identified areas where they felt evidence guiding dietary advice was lacking. For UK participants specifically, this related to guidance surrounding nutritional supplements and high output stoma management. Ileostomates emphasised the need for individualised advice, recognising that dietary tolerance and nutritional requirements vary, and the challenges that this presents for HCPs delivering support. Overall, HCPs shared this view that there is a lack of clinical evidence for dietary management strategies in ileostomy and so, *building the evidence base* was a priority for many in improving their practice.

**Table 4:** Summary of categories, subcategories and illustrative quotes relating to institutional and public policy factors

Ileostomate		HCP	
Embedding continuity in healthcare systems		Embedding continuity in healthcare systems	
*Early education & continued follow up* UK, AUS	*“So*,* I kind of felt a little bit lost*,* I guess going into the surgery because I kind of didn’t know what would be expected. Later people were saying to me*,* oh*,* will you to be able to do this?… I was like*,* I don’t know actually.”* [S25, AUS]	*Early education & continued follow-up* UK, AUS	*“You need to be seeing them kind of every 4 to 6 weeks for those first couple of months*,* just so that you can see them regularly*,* like build their rapport like they would have with their stoma nurses*,* and try and g*et *them to incorporate things in.”* [H01, dietitian, UK]
*Signposting* UK	*“So*,* I think the communication around it is what’s lacking. The information is there… but how that information is then communicated is*,* is quite poor.”* [S12, UK]	*Managing caseload capacity within system constraints* UK, AUS	*“So*,* I*,* I think that’s where*,* a lot of the difference is between here and the NHS. It’s communication. It’s money because it’s double ended service here. You’ve got private and you’ve got public… And like burnout is massive in the NHS*,* and I don’t see as much of that here as I did at home.”* [H18, dietitian, AUS]
*Trusted & comprehensive information* AUS	*“But what I found was I*,* I had to run around to build a comprehensive picture and and*,* and I think that’s lacking out there on the web… if there was a central site that had bought that together… that would have saved a lot of time.”* [S17, AUS]	*Shortage of specialist services* AUS	*“It’s like people are just having really big issues with little*,* little things that could be easily fixed if it was just an easier system to manage. We’re touching the*,* like*,* very minimal top of the people that we’re seeing because that’s all there is capacity for.”* [H19, dietitian, AUS]
Addressing gaps in knowledge and evidence		Addressing gaps in knowledge and evidence	
*Improving HCP knowledge* UK, AUS	*“I think there are a lot of gaps in the knowledge…And I found that even when I’ve asked professional people… I’ve never felt that they’ve really got a good grasp of what they need to be able to tell you.”* [S20, AUS] *“So*,* I think that’s probably a gap that I see*,* just maybe the need for more tailored information because everybody is different.”* [S05, UK] *“There’s no explanation… there was no rhyme or reason.”* [S22, AUS]	*Improving HCP knowledge* UK, AUS	*“It’s every day. I’m always learning things…”* [H10, SCN, UK]. *“Other health professionals… or more junior dietitians would be more likely just to say we need to follow all this and and being incredibly strict about quite an anecdotal approach.”* [H12, dietitian, UK] *“Our junior doctors will tell people with a high output that they just need to drink more…and we’re like*,* no*,* don’t tell them that.”* [H20, SCN, AUS]
*Building the evidence base* UK, AUS	*“Some people can eat stuff*,* some people can’t…. I don’t know how a dietitian can come and work out what’s right for you*,* because you’re gonna be different maybe to the person in the bed next to you.”* [S27, AUS] *“I kind of struggle with the fact that this*,* you know*,* in this day and age and the marvels of medicine*,* that there’s not*,* there’s not something that sort of can be done… the most… is like loperamide and Saint Marks and that’s it.”* [S06, UK]	*Building the evidence base* UK, AUS	*“I just wonder*,* would would two weeks* [on a low fibre diet] *be enough for most people… Are you just limiting people for no reason?”* [H06, dietitian, UK] *“I worked out over the years that we don’t see people come back with obstructions. And if that’s the reason we tell them not to eat these things*,* well*,* there’s no evidence really.”* [H13, SCN, AUS] *“It kind of drives me insane that that’s a recommendation…. It’s like no one should be eating marshmallows…*,* the gelatine may help thicken it up*,* but it’s like you have to eat a lot of it and it’s just sugar.”* [H20, SCN, AUS]

HCP, health care professional; SCN, stoma care nurse; AUS, Australia

## Discussion

This study compared ileostomates’ experiences of dietary advice across the UK and Australia, integrating insights from HCPs across both countries to provide a holistic view of dietary practices. Mapping categories to the SEM produced a comprehensive view of patient experience and complementary pathways for dietary support, ranging from the underlying condition which resulted in surgery (ileostomate) and initial referral (HCP), to consideration of the shortcomings of the available dietary support within and without the healthcare system.

Formation of an ileostomy represents a major life change, with alterations to dietary intake central to postop recovery [25]. Extending evidence of reduced overall and diet-related QoL [26], current findings illustrate how social challenges and altered eating behaviours reduce enjoyment and confidence around eating. Fear of food bolus blockage emerged as a prominent driver of dietary behaviour in both ileostomate groups, supporting earlier work identifying blockages as a key motivator for sustained food avoidance [9, 11]. While only a small number of participants described experiences of postop ileus, these accounts suggest it may undermine dietary reintroduction and promote cautious eating behaviours, indicating a potential need for targeted dietary support.

Food avoidance was ubiquitous in UK and Australian participants, consistent with reports of altered dietary patterns after ileostomy and long-term restriction of high fibre foods [10]. HCPs raised concerns surrounding such patterns and noted that clinical evidence for dietary management remains limited, contributing to reliance on anecdotal reports from patients and clinical experience [27]. Current findings demonstrate how this leads to the variations in practice, inconsistent messaging and ultimately confusion for patients and HCPs reported in previous qualitative and observational data [8, 28]. Importantly, the current study suggests precautionary restriction may reinforce dietary avoidance in our sample of ileostomates (8.3 ± 8.0 years postop), echoing previous work which report as many as 60% of ileostomates restrict foods, such as fruit and vegetables, for more than a year after their initial surgery because they were advised to do so [9]. HCPs also expressed uncertainty regarding the strength of evidence underpinning low fibre and other dietary advice, highlighting variation in practice and the need to strengthen the evidence base for dietary guidance.

Variability in dietary tolerance was evident; while ileostomates described differing levels of food tolerance, HCPs reflected on the limitations of standardised guidance, highlighting challenges in applying ‘best practice’ advice, reinforcing the need for individualised support. Tailored nutritional guidance can reduce ileostomy related symptoms by ~ 48% [29], and improve nutritional status and intake [30, 31]. Combined perspectives from the current study indicates that patients would benefit from early education and continued follow up with a SCN or dietitian; an approach consistent with the European Crohn’s and Colitis Organisation consensus position that all patients with IBD should have access to an experienced dietitian [32]. However, these expectations highlight a central tension: personalised care and consistent messaging are both essential, yet the provision of dietary support is increasingly difficult to achieve within existing system constraints.

Postoperative dietary experiences were shaped by the hospital environment. Participants reported negative perceptions of inpatient meals, a factor previously linked to a 40% increased risk of complications and a 90% increase in delayed discharge [33]. Although improvements to hospital food require system level action, this study identified support from family as a key facilitator to dietary reintroduction during inpatient admission and long-term. Evidence that family-centred interventions and peer-peer support can enhance QoL among stoma patients further underscores this point [34, 35]. Taken, together, these findings highlight the value of incorporating wider support networks in future strategies to improve delivery and implementation of dietary advice in ileostomates. From the HCP perspective at the systemic level, consistent dietary support was limited by staffing, clinic times, and varied caseloads, reflecting financial constraints across healthcare systems [17].

Australian ileostomates emphasised hydration and increased stoma output, the latter of which is strongly linked to pouch leakage and PSCs [36]. The relative prominence of these concerns among Australian participants is likely reflective of challenges in accessing specialist care for ongoing ileostomy-related complications in regional and remote areas. In contrast, UK ileostomates recognised hydration concerns at wider systems level, citing gaps in evidence and guidance. Although comparative data are limited, European evidence suggests UK clinicians place less emphasis on hydration than those in warmer regions [37]. The heightened attention of hydration among Australian participants may therefore reflect differences in climate, patient education, or a combination of the two. Overall, these findings highlight how structural and geographic factors shape access to care and patients’ dietary concerns.

Finally, where access to and continuity of care was limited, many ileostomates sought support from online forums. Whilst HCPs cautioned use of these sources due to potential for misinformation, echoing Rhys-Jones et *al.* [38] who described online information for patients with an ileoanal pouch as “poor quality,” it also indicates acceptability of remote support. Evidence from other cohorts suggests digital and telehealth interventions can improve gastrointestinal symptoms [39, 40], and ease caseload burden for HCPs. Such methods could be readily adapted and implemented within an ileostomate population.

### Strengths and limitations

To our knowledge, this is the first study to compare patient and HCP experiences and needs in relation to dietary management of ileostomy. Inclusion of participants from the UK and Australia allowed cross-national comparisons within two distinct healthcare systems. A key strength was the large and diverse cohort of HCP and ileostomate participants, enhancing breadth and transferability of findings. Inclusion of both patients and HCPs aligned with the SEM, providing a multilevel understanding of dietary experiences that considers intrapersonal, interpersonal and community, and institutional and public policy factors. Limitations include recruitment via social media, potentially biasing towards proactive, information seeking patients, and inclusion of SCNs and dietitians already engaged in ileostomy care indicating a pre-existing interest in nutrition (SCNs) or stoma care (dietitians).

### Implications for practice

The findings from this study have significant implications for clinical practice, highlighting the need to move beyond a ‘one size fits all’ approach, towards evidence-based and person-centred guidance. Development and institutional implementation of consensus dietary guidelines should be prioritised to reduce variation in professional practice and improve confidence amongst ileostomates. Critical evidence gaps have been identified, including efficacy of the low fibre diet, dietary strategies for symptom management, and long-term effects of dietary restriction. Addressing these evidentiary gaps should take precedence when designing future studies.

## Conclusion

This research illustrates the complex multilevel factors shaping ileostomates’ dietary experiences and the related challenges perceived by health professionals. Applying the social ecological model illustrated how health system constraints influence care. Findings reveal gaps in follow-up support and inconsistent guidance, underscoring the need for consensus dietary guidelines and greater use of digital and telehealth to improve accessibility of long-term care and reduce caseload burden for professionals.

## Supplementary Information

Below is the link to the electronic supplementary material.

Supplementary Material 1

## Data Availability

The data that support the findings of this study are not openly available due to reasons of sensitivity and are available from the corresponding author upon reasonable request.
