# Supplementary material for: Diet and ileostomy: a qualitative comparison of patient and healthcare professional perspectives in the United Kingdom and Australia
Source: Eur J Nutr. 2026 Jun 15;65(5):212. doi: 10.1007/s00394-026-04036-1 (PMC13364819; doi:10.1007/s00394-026-04036-1)
Supplement: Supplementary file 1 — Supplementary Material 1 [file 394_2026_4036_MOESM1_ESM.docx]

| **Table S1: Semi structured topic guides for ileostomate and HCP interviews** | | |
| --- | --- | --- |
| **Structure** | **Topic(s)** | **SEM level(s)** |
| ***Ileostomate group*** | |  |
| *Pre-operative* | Reasons for ileostomy; underlying condition(s)  Emergency or elective surgery  Dietary intake & advice received during this period | Intrapersonal |
| *Post-operative (acute)* | Source(s) / type of dietary advice; verbal or print resources  Practicality of advice; ease of implementation  Impact of diet (advice) on post-operative recovery | Intrapersonal  Interpersonal & Community |
| *Post-operative (long-term)* | Accessibility of advice; source(s) / type of dietary advice  Practicality of advice; ease of implementation  Impact of diet (advice) on life with an ileostomy | Intrapersonal  Interpersonal & Community |
| *Future considerations* | Suggestions to improve provision of dietary advice | Institutional & Public Policy |
| ***HCP group*** | | |
| *Professional practice* | Standard process for patient assessment & review(s)  Timeframe; pre-, post-operative & post-discharge assessment(s)  Availability & use of dietary resources | Institutional & Public Policy |
| *Challenges for practice* | Barriers to provision of dietary support; pre-, post-operative & long-term  Clinical challenges | Intrapersonal  Interpersonal & Community  Institutional & Public Policy |
| *Patient experience* | Challenges for patients; pre-, post-operative & long-term  Impact of diet on patients’ management of their ileostomy | Intrapersonal  Interpersonal & Community |
| *Future considerations* | Suggestions to improve provision of dietary advice or professional practice | Institutional & Public Policy |

**Table S2: Prevalence of (sub)categories across ileostomate and HCP participant groups**

| **Category** | **Subcategory** | **Ileostomate** | | **HCP** | |
| --- | --- | --- | --- | --- | --- |
|  |  | **UK (*n*=13)** | **AUS (*n*=13)** | **UK (*n*=12)** | **AUS (*n*=12)** |
| *Dietary & lifestyle adaptations* | *Dietary restriction* | 13 (100.0) | 13 (100.0) | 11 (91.7) | 12 (100.0) |
|  | *Self-directed trial & error* | 13 (100.0) | 13 (100.0) | - | - |
|  | *Living with dietary change* | 13 (100.0) | 12 (92.3) | - | - |
|  | *Symptom management* | 12 (92.3) | - | - | - |
|  | *Output & hydration* | - | 13 (100.0) | - | - |
|  | *Providing support during a major life change* | - | - | 9 (75.0) | 11 (91.7) |
|  | *Fear & uncertainty* | - | - | 10 (83.3) | 10 (83.3) |
|  | *Patient understanding* | - | - | 12 (100.0) | 12 (100.0) |
| *Clinical needs & individual variability* | *Need for surgery* | 10 (76.9) | 13 (100.0) | - | - |
|  | *Surgical recovery & post-op complications* | 10 (76.9) | 10 (76.9) | - | - |
|  | *Undernutrition & weight management* | 11 (84.6) | 10 (76.9) | - | - |
|  | *Special dietary requirements* | - | 3 (23.1) | - | - |
|  | *Pouch leakage & PSCs* | - | 6 (46.2) | - | - |
| *Delivering person centred care in practice* | *Integrating needs* | - | - | 12 (100.0) | 11 (91.7) |
|  | *Reassurance & reinforcement of advice* | - | - | 11 (91.7) | - |
|  | *Patient-led stepwise support* | - | - | - | 11 (91.7) |
| *Experiences of support within & beyond clinical care*  *Community support and system level collaboration* | *Value of HCP support* | 10 (76.9) | 11 (84.6) | - | - |
|  | *Geographical barriers* | - | 5 (38.5) | - | 5 (41.7) |
|  | *Peer experience & online forums* | 12 (92.3) | 13 (100.0) | 6 (50.0) | - |
|  | *Family support* | 4 (30.7) | 9 (69.2) | 5 (41.7) | - |
|  | *Interprofessional working* | - | - | 12 (100.0) | 12 (100.0) |
|  | *Importance of consistent messaging* | - | - | 10 (83.3) | 11 (91.7) |
|  | *Provision of hospital food* | - | - | 7 (58.3) | 5 (41.7) |
| *Embedding continuity in healthcare systems* | *Early education & continued follow up* | 11 (84.6) | 10 (76.9) | 12 (100.0) | 12 (100.0) |
|  | *Signposting* | 6 (46.2) | - | - | - |
|  | *Trusted & comprehensive information* | - | 8 (61.5) | - | - |
|  | *Managing caseload capacity within system constraints* | - | - | 10 (83.3) | 9 (75.0) |
|  | *Shortage of specialist services* | - | - | - | 5 (41.7) |
| *Addressing gaps in knowledge and evidence* | *Improving HCP knowledge* | 4 (30.7) | 8 (61.5) | 11 (91.7) | 12 (100.0) |
|  | *Building the evidence base* | 7 (58.3) | 8 (61.5) | 9 (75.0) | 8 (66.7) |

HCP, healthcare professional; AUS, Australia; PSCs, peristomal skin complication.

Data presented as n (%)
